# Reconfiguration of static and dynamic thalamo-cortical network functional connectivity of epileptic children with generalized tonic-clonic seizures

**DOI:** 10.3389/fnins.2022.953356

**Published:** 2022-07-22

**Authors:** Yongxin Li, Jianping Wang, Xiao Wang, Qian Chen, Bing Qin, Jiaxu Chen

**Affiliations:** ^1^Guangzhou Key Laboratory of Formula-Pattern of Traditional Chinese Medicine, Formula-pattern Research Center, School of Traditional Chinese Medicine, Jinan University, Guangzhou, China; ^2^The Second Affiliated Hospital of Guangzhou Medical University, Guangzhou, China; ^3^Epilepsy Center and Department of Neurosurgery, The First Affiliated Hospital, Jinan University, Guangzhou, China; ^4^Department of Pediatric Neurosurgery, Shenzhen Children’s Hospital, Shenzhen, China

**Keywords:** generalized tonic-clonic seizure, epileptic children, resting-state fMRI, thalamus, temporal variability, dynamic functional connectivity, dynamic effective connectivity

## Abstract

**Objective:**

A number of studies in adults and children with generalized tonic-clonic seizure (GTCS) have reported the alterations in morphometry, functional activity, and functional connectivity (FC) in the thalamus. However, the neural mechanisms underlying the alterations in the thalamus of patients with GTCS are not well understood, particularly in children. The aim of the current study was to explore the temporal properties of functional pathways connecting thalamus in children with GTCS.

**Methods:**

Here, we recruited 24 children with GTCS and 36 age-matched healthy controls. Static and dynamic FC approaches were used to evaluate alterations in the temporal variability of thalamo-cortical networks in children with GTCS. The dynamic effective connectivity (dEC) method was also used to evaluate the directions of the fluctuations in effective connectivity. In addition, the relationships between the dynamic properties and clinical features were assessed.

**Results:**

The static FC analysis presented significantly decreased connectivity patterns between the bilateral thalamus and between the thalamus and right inferior temporal gyrus. The dynamic connectivity analysis found decreased FC variability in the thalamo-cortical network of children with epilepsy. Dynamic EC analyses identified increased connectivity variability from the frontal gyrus to the bilateral thalamus, and decreased connectivity variability from the right thalamus to the left thalamus and from the right thalamus to the right superior parietal lobe. In addition, correlation analysis revealed that both static FC and connectivity temporal variability in the thalamo-cortical network related to the clinical features (epilepsy duration and epilepsy onset time).

**Significance:**

Our findings of both increased and decreased connectivity variability in the thalamo-cortical network imply a dynamic restructuring of the functional pathways connecting the thalamus in children with GTCS. These alterations in static and temporal dynamic pathways connecting the bilateral thalamus may extend our understanding of the neural mechanisms underlying the GTCS in children.

## Introduction

Generalized tonic-clonic seizure (GTCS) is a subgroup of idiopathic generalized epilepsy that is typically characterized by generalized spike-wave discharges (2.5-5 Hz) (Ji et al., 2014). The clinical symptoms of GTCS mainly include muscle contraction of the body and complete loss of consciousness. Patients with GTCS showed cognitive impairments in their attention, memory, and executive function (Hommet et al., 2006). One of the hallmarks of GTCS is the absence of visible abnormalities on routine magnetic resonance imaging (MRI). Based on the connectome view, the GTCS has been hypothesized as a disorder of brain connectivity (Kim et al., 2014; Li et al., 2020a; Royer et al., 2022). It has been widely accepted that generalized spike-wave discharges might be caused by the imbalance of local excitation and inhibition through thalamo-cortical network (Wang et al., 2012; Lüttjohann and van Luijtelaar, 2022). As a result, abnormal activity was detected in wide brain regions in patients with epilepsy (Assenza et al., 2020).

As we know, the thalamus is globally connected with distributed cortical regions. Previous studies have found that the thalamus is a critical hub region that is involved in the integration of information across the cortical networks (Hwang et al., 2017; Elvsåshagen et al., 2021). The thalamus plays a central role in ongoing cortical functioning, which is performed by the thalamo-cortical network. Neuroimaging studies in epilepsy have found that low volume of the thalamus is a common pattern across epilepsy syndromes (Whelan et al., 2018; Xu et al., 2021). Thalamic atrophy may be the effect of seizure activity, such as thalamo-cortical network remodeling or thalamic disconnection (Bernhardt et al., 2009; Li et al., 2017b; Chen et al., 2019). Previous studies have also detected that atrophy of the thalamic nuclei and resting-state functional hyperconnectivity between the thalamus and cerebral cortex can be considered as imaging markers in generalized patients with refractory epilepsy (Wang et al., 2018; Chen et al., 2021). Specific changes in the thalamus imply that this region plays an important role in epilepsy. A recent study has detected that abnormal functional and structural integration in the cerebellum, basal ganglia, and thalamus could result in an imbalance of inhibition and excitability in the brain system of idiopathic generalized epilepsy (Gong et al., 2021). Patients with GTCS showed a more constrained network embedding of the thalamus and an increased functional diversity of the frontocentral neocortical regions (Wang et al., 2019a). Graph theory analysis of the structural covariance network of the gray matter found that children with GTCS also showed significant alterations in the nodal betweenness in the right thalamus, bilateral temporal pole, and some regions of DMN (Li et al., 2020b). These previous neuroimaging studies have provided converging evidence for both intrinsic functional connectivity (FC) and structural connectivity abnormalities of the thalamo-cortical network in patients with GTCS (Kim et al., 2014; Lee et al., 2014; Ji et al., 2015; Li et al., 2016; Wang et al., 2019a; Gong et al., 2021). Focus on the functional and structural changes of thalamo-cortical network would provide additional information to understand the neural mechanism of GTCS.

Although these basic connectivity approaches are widely used in previous epilepsy studies and improved our understanding of GTCS, these methods may not be sufficient to fully characterize the specific role of the thalamus in patients with GTCS. How the organization of thalamo-cortical network was interfered by the seizure and the temporality and causality between the epilepsy-related regions were not fully understood. Currently, there are two directions of studies trying to solve the above concerns. One direction is based on the deep brain stimulation that aims to verify the functional role of the thalamus during the treatment process. Recent studies have confirmed the satisfactory results of thalamic nucleus deep brain stimulation in drug-resistant generalized epilepsy (Fasano et al., 2021; Vetkas et al., 2022). This approach is effective in clinical practice but is invasive. Another direction is based on the dynamic connectivity methods that aim to explore the spontaneous fluctuations in the activity and connectivity with the thalamus in epilepsy. The conventional FC analysis assumes that the communication between regions is relatively stable during the entire scan. This method may not capture the dynamic nature of communication. The human brain is a complex dynamic system (Fox et al., 2005). Recently, a dynamic FC (dFC) method was developed by measuring the variability in the strength or spatial dynamic organization of brain connectivity (Preti et al., 2017; Rolls et al., 2021). Previous studies in neurological disorders have proved that this approach can sensitively capture the time-varying changes of the ongoing activity over the whole scan time (Liao et al., 2018; Kottaram et al., 2019; Guo et al., 2020). In epilepsy, compared to the conventional FC analysis, dynamic techniques can be used to identify additionally activated brain regions during the course of interictal epileptic discharges (Kowalczyk et al., 2020). Dynamic FC analyses have demonstrated that state transitions and modular function of dissociable hippocampal networks were altered in temporal lobe epilepsy, which can reflect different memory phenotypes (Li et al., 2022a). Disruption of this dynamic organization may be the neuroimaging expression of the cognitive dysfunction in epilepsy patients. For adults with GTCS, state-specific dFC disruptions and the majority of aberrant functional connectivity were observed in DMN (Liu et al., 2017). Recently, dFC approaches were also used to evaluate alterations in the temporal variability of FC in patients with GTCS at the region and network levels (Jia et al., 2020; Li et al., 2022c). Patients with GTCS showed a dynamic restructuring of the large-scale brain networks. Although these previous studies offered evidence for the dynamic interaction among whole-brain functional networks in adults with GTCS, no study regarding GTCS has been performed to investigate the temporal variability of FC with the thalamus and their connectivity direction in thalamo-cortical network. Investigation of brain dynamic FC network and dynamic effective connectivity (dEC) in thalamo-cortical network could allow us to understand the dynamic roles of the thalamus in the brain with GTCS.

Additionally, most of these previous studies were performed in adults with GTCS (Wang et al., 2011; Ji et al., 2014; Li et al., 2016; Liu et al., 2017; Jia et al., 2020). Children with GTCS have not received enough attention. Up to now, only a few neuroimaging studies were conducted in children with GTCS (Wang et al., 2018; Li et al., 2020a,b, 2022b). In these previous studies in children with GTCS, brain activity, gray matter volume, and the topological properties of the brain network were analyzed. The consistent results among these studies demonstrate that children with GTCS showed both functional and structural abnormalities in the thalamus and DMN. Although these previous studies indicate that the thalamus also plays an important role in children with GTCS, the dynamic exchange of information in the thalamo-cortical network remains largely unknown. Recently, the temporal variability in FC and EC has attracted increasing attention in epilepsy to understand the dynamic role of the regions in the brain system (Jia et al., 2020; Kowalczyk et al., 2020). Thus, examining the temporal properties of the thalamo-cortical network in children with GTCS would provide further information for understanding this disease.

In the present study, we aim to explore the temporal variability of the thalamo-cortical network connectivity in children with GTCS. We first used the static FC based on the bilateral thalamus to obtain the specific connectivity changes in children with GTCS. Then, we conducted dFC and dEC analysis to investigate the between-group differences in temporal variability and connectivity direction in thalamo-cortical network. Finally, the correlation between altered connectivity and clinical variables was measured in children with GTCS. On the basis of the previous studies in GTCS, we hypothesized that dynamic FC and dynamic EC of the thalamo-cortical network would be reconfigured in children with GTCS compared with the healthy controls. Variability of the connectivity in the thalamo-cortical networks should be responsible for the clinical characteristics.

## Methods

### Subjects

This study recruited 24 children with GTCS (9 female, mean age: 69.94 ± 46.36 months) from the Shenzhen Children’s Hospital. All human procedures were approved by the Ethical Committee of the Shenzhen Children’s Hospital. All participants’ parents or their guardians provided written informed assent and consent. Based on their clinical and seizure semiology information, all patients were diagnosed to have genetic associated epilepsy with GTCS by two experienced neurologists (Fisher et al., 2017). The inclusion criteria for patients were as follows: (1) symmetrical tonic and clonic seizure, and loss of consciousness during seizure without any other focal features; (2) typically showed generalized spike-wave or poly-spike-wave without any other focal discharge on EEG; (3) no focal abnormality was detected in all patients on routine MRI; and (4) no other developmental disability. The exclusion criteria included the following: (1) pathologic abnormality on conventional MRI; (2) history of addition or neurological diseases besides epilepsy; (3) subjects with MRI contraindications; (4) age older than 13 years; and (5) head motion exceeding 3 mm in translation or 3° in rotation. All patients took at least one antiepileptic drug (AED: topiramate, valproic acid, levetiracetam, or oxcarbazepine) to control seizures before the image scanning. Eleven patients took one AED, 10 patients took two AEDs, and 3 patients took three AEDs. All patients were maintained seizure-free for at least 2 days prior to the MRI scanning. Thirty-six healthy controls (HCs, 11 female, mean age: 71.89 ± 31.13 months) were enrolled. All control children did not have a history of neurological disorders or psychiatric illnesses or gross abnormalities on brain MRI. In order to reduce the head movement during the MRI scanning, 18 children (9 controls and 9 patients) under the age of 4 years were sedated with 10% chloral hydrate (dosage: 50 mg/kg, the maximum dose was 1 g). The demographic and clinical information of the two groups is listed in Table 1.

**TABLE 1:** Demographic and clinical information data of the subjects.

Characteristics	Patient group (Mean ± SD)	Control group (Mean ± SD)	Comparisons
Sex (female/male)	9/15	11/25	*X*^2^ = 0.31 (*p* = 0.58)
Age (month)	69.94 ± 46.36	71.89 ± 31.13	*t* = 0.20 (*p* = 0.85)
Disease onset age (month)	37.35 ± 46.22	\	
Epilepsy duration (month)	32.58 ± 31.20	\	

### Scan acquisition

All MRI data were acquired with a 3.0T German Siemens Trio Tim scanner (MAGNETOM, Germany, 8-channel prototype quadrature birdcage head coil) at the Shenzhen Children’s Hospital, Shenzhen, China. During the imaging scanning, the head of all the participants was fixed with foam padding to minimize head movements. Earplugs were also used to reduce the impact of machine noise. All participants were lying quietly, as motionless as possible. During the imaging scanning, the participants over the age of 4 years were instructed to keep still with their eyes closed, remain awake, and instructed not to think about anything. We observed them throughout the whole scanning process, and enquired about their conditions after the test. They were asked whether they fell asleep or moved their head during the scanning process. The acquisition parameters for the resting-state fMRI data of all subjects were as follows: repetition time (TR) = 2,000 ms, echo time (TE) = 30 ms, flip angle = 90°, field of view (FOV) = 220 × 220 mm^2^, 94 × 94 matrix, slice thickness = 3 mm, and 36 interleaved axial slices. A total of 130 volumes were obtained in each run. High-resolution 3D T1-weighted anatomical images were acquired for all the subjects in the sagittal orientation using a MPRAGE sequence: TR = 2,300 ms, TE = 2.26 ms, flip angle = 8°, FOV = 200 × 256 mm^2^, 200 × 256 matrix, slice thickness = 1 mm, and 160 sagittal slices.

### Image data preprocessing

Functional images were preprocessed using the data assistant software DPABI (Yan et al., 2016). The following steps were performed in preprocessing stage: (1) The first 10 scans were discarded to allow for magnetization equilibrium. (2) We performed slice timing and motion correction for the remaining images. The translations in each direction and the rotations in angular motions were estimated in this step. The participants with a head motion of >3 mm in maximum displacement or >3° rotation during data acquisition were excluded from the study. (4) The mean frame-wise displacement (FD) was computed by averaging the FD of each participant across the time points, which can determine the comparability of head movement across groups. No significant differences were found between the two groups (HCs: mean FD = 0.145 ± 0.113 mm, children with GTCS: mean FD = 0.131 ± 0.087 mm). (5) Individual 3D T1-weighted images were co-registered to the mean functional images by rigid body transformation after the motion correction. The transformed structural images were then segmented into gray matter, white matter, and cerebrospinal fluid by using a unified segmentation algorithm. (6) The segmented images were normalized to the Montreal Neurological Institute (MNI) space by using a 12-parameter non-linear transformation. The obtained transformation parameters were then applied to the functional images, and these functional images were normalized to the MNI space. (7) The normalized images were resampled into a voxel size of 3 mm × 3 mm × 3 mm and spatially smoothed with a half-maximum Gaussian kernel of 6 mm full width. (8) Linear regression analysis was used to control for confounding factors, including Friston-24 motion parameters, white matter signals, and cerebrospinal fluid signals. (9) Finally, band-pass temporal filtering (0.01–0.08 Hz) was used to remove the effects of very-low-frequency drift and high-frequency noise (Biswal et al., 1995; Lowe et al., 1998).

### Static functional connectivity analysis

The FC maps were obtained using the voxel-wise approach by computing FC between the region of interest (ROI) and each voxel within the brain. The bilateral thalamus was used as the seed ROIs, which were obtained from the Anatomical Automatic Labeling atlas. The time series of the voxel in each ROI was extracted and averaged, followed by a correlation with the time series of each other voxel across the entire brain. The correlation value was z-transformed for each sample. The *z*-transformed FC maps were compared between the two groups by a two-sample *t*-test (AlphaSim corrected for multiple comparisons; *p* < 0.005)^1^. In this step, age and sex were regressed as confounding covariates.

### Dynamic functional network connectivity

To identify the dFC variability of the thalamo-cortical network, we used a sliding window dFC approach in the Dynamic BC toolbox (Liao et al., 2014) ^2^. Currently, window length is an open area of research in the sliding window-based dFC analysis. This parameter is important for capturing the rapidly shifting relationship between the windows. Here, a rectangle window with a size of 60 s (30 TRs) was selected to segment the resting-state time series by sliding across the whole scan with a step of 1 TR, resulting in 90 overlapping windows per subject. We chose the window length of 60 s because previous studies have proven that the window size of around 30-60 s could optimize the balance between the temporal resolution and the quality of the functional network connectivity estimate (Allen et al., 2014). We also tried other window lengths (20 TRs) to further examine the possible effects on dFC results. In each sliding window, we computed the temporal correlation coefficient between the truncated time course of the thalamus seeds and those of all the other voxels. After this step, a set of sliding window correlation maps were created for each participant. All the correlation maps were transformed by a Fisher’s *r*-to-*z* transformation to improve the normality of the correlation distribution. The variance in the time series of the correlation coefficient was computed by calculating the standard deviation of *z*-values at each voxel to assess the dFC variability.

To examine the difference in dFC variability patterns between the two groups, a two-sample *t*-test analysis was performed on the standard deviation in *z*-values at each voxel. In this step, age and sex were regressed as confounding covariates. The statistical significance level for the comparison analysis was thresholded at *p* < 0.005, after AlphaSim correction.

### Dynamic effective network connectivity

Seed-based dEC analysis was obtained using time-varying dynamic Granger causality. The time-varying strength and direction of the connections were calculated between the thalamus and each voxel within the brain. We used a sliding window dEC approach in the Dynamic BC toolbox. Here, a rectangle window with a size of 60 s (30 TRs) was selected to segment the resting-state time series by sliding across the whole scan with a step of 1 TR, resulting in 90 overlapping windows per subject. We also tried other window lengths (20 TRs) to further examine the possible effects on dEC results. In each sliding window, we computed the strength and direction of the connections between the truncated time course of the thalamus seeds and those of all the other voxels. After this step, two sets of sliding window correlation maps were created for each participant: one represents the input directions and the other represents the output directions. All the correlation maps were transformed by a Fisher’s r-to-z transformation to improve the normality of the correlation distribution. The variance in the time series of the correlation coefficient was computed by calculating the standard deviation of *z*-values at each voxel to assess the dEC variability.

To examine the difference in dEC variability patterns between the two groups, a two-sample *t*-test analysis was performed on the standard deviation in *z*-values at each voxel. In this step, age and sex were regressed as confounding covariates. The statistical significance level for the comparison analysis was thresholded at *p* < 0.005, after AlphaSim correction.

### Clinical correlation analysis

We then further explored the potential relationship between the brain static FC and clinical characteristics (epilepsy duration and epilepsy onset age) in children with GTCS. Partial correlation analyses between the connectivity variability (dFC variability and dEC variability) and clinical characteristics (epilepsy duration and epilepsy onset age) were conducted in children with GTCS after controlling for age and sex. The statistical significance level for the correlation analysis was set at *p* < 0.05.

## Results

### Demographic characteristics

The demographic and clinic characteristics of the children with GTCS and controls are listed in Table 1. No significant differences were found (*p* > 0.05) in age or sex distribution between the two groups. The mean epilepsy duration (32.58 ± 31.20 months) and disease onset age (37.35 ± 46.22) of the children with GTCS were also collected and listed in Table 1.

### Differences in functional connectivity analysis

**FIGURE 1:**
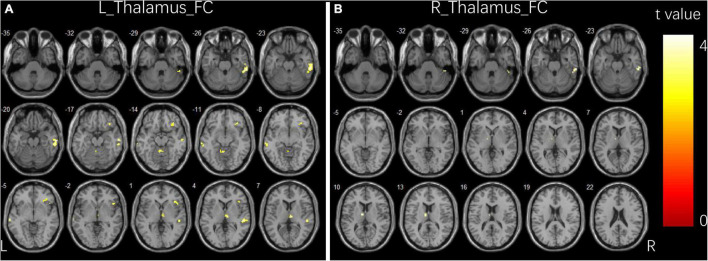
Group differences in functional connectivity for bilateral thalamus seeds between two groups. **(A)** Brain regions showing significant differences in FC for left thalamus seed. **(B)** Brain regions showing significant differences in FC for right thalamus seed. The comparison analysis was thresholded at *p* < 0.005, AlphaSim corrected. R, right hemisphere; L, left hemisphere; FC, functional connectivity.

Compared to controls, children with GTCS showed a significant decrease in FC connecting the bilateral thalamus. The connectivity pattern of the left thalamus was centered on the right inferior temporal gyrus (ITG), right thalamus, bilateral middle temporal gyrus (MTG), right orbital inferior frontal gyrus (IFG), and right precuneus (Figure 1A). The connectivity pattern of the right thalamus was centered on the right ITG and left thalamus (Figure 1B). The details showing significant differences between the groups are listed in Table 2.

**TABLE 2:** Significant group differences in FC analysis.

Cluster location	Statistical values		Peak (MNI)		
	Cluster size	*t-value*	*x*	*y*	*z*
**Seed L thalamus Control > Patient**					
R inferior temporal gyrus	87	3.86	60	−33	−27
R middle temporal gyrus		3.46	66	−18	−21
R superior temporal gyrus	21	3.73	54	−27	3
L middle temporal gyrus	21	3.34	−66	−24	−6
R thalamus	23	3.28	6	−18	6
R orbital inferior frontal gyrus	25	3.25	36	30	−15
R precuneus^#^	11	3.2	6	−51	21
**Seed R thalamus Control > Patient**					
R inferior temporal gyrus	22	3.28	60	−33	−27
L thalamus	15	3.35	−6	−9	12

The MNI coordinates and t-values for the FC results. Threshold for significant clusters reported here was set at p < 0.005 (AlphaSim correction) and cluster size of 14.

^#^The result was uncorrected; MNI, Montreal Neurological Institute.

### Dynamic functional connectivity variability results

**FIGURE 2:**
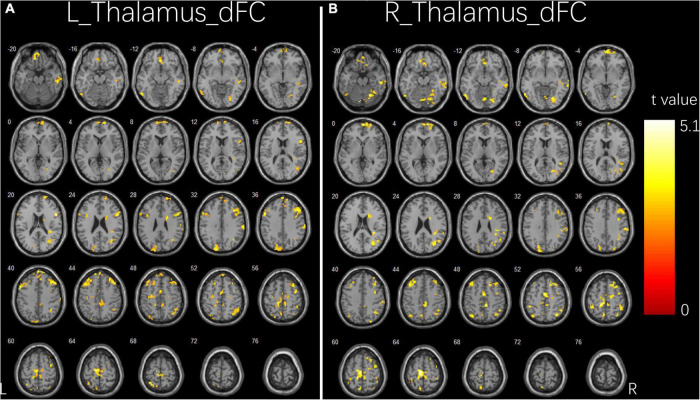
Group differences in dFC variability for bilateral thalamus seeds between two groups. **(A)** Brain regions showing significant differences in dFC variability for left thalamus seed. **(B)** Brain regions showing significant differences in dFC variability for right thalamus seed. The comparison analysis was thresholded at *p* < 0.005, AlphaSim corrected. R, right hemisphere; L, left hemisphere; dFC, dynamic functional connectivity.

Figure 2 and Table 3 illustrate the significant differences in dFC variability between the two groups for the bilateral thalamus seeds. Compared with the HCs, the patient group exhibited a significantly less dFC variability between the left thalamus seed and regions of bilateral IFG, right middle frontal gyrus (MFG), right angular gyrus, right inferior parietal lobule (IPL), right fusiform, right ITG, right cerebellum, left precentral gyrus, left supplementary motor area (SMA), left superior parietal lobule (SPL), left precuneus, and left paracentral gyrus (Figure 2A). Compared with the HCs, the patient group exhibited significant less dFC variability between the right thalamus seed and regions of bilateral MFG, bilateral IPL, right superior frontal gyrus (SFG), right angular gyrus, right SMA, right SPL, right fusiform, right ITG, right MTG, right cerebellum, left precuneus, and left paracentral gyrus (Figure 2B). No significant excessive dFC variability was found in the patient group with the bilateral thalamus seeds.

**TABLE 3:** Significant group differences in dFC analysis.

Cluster location	Statistical values		Peak (MNI)		
	Cluster size	*t*-value	*x*	*y*	*z*
**Seed L thalamus Control > Patient**					
R opercular inferior frontal gyurs	270	5.1	54	12	21
R middle frontal gyrus		4.37	33	39	45
L paracentral lobe	261	4.93	−12	−15	66
L precentral gyrus	188	4.69	−54	12	39
L middle frontal gyrus		4.03	−39	21	45
L superior frontal gyrus	117	4.62	−15	54	42
R superior frontal gyrus		4	15	57	39
L superior parietal lobule	35	4.27	−24	−60	69
L orbital inferior frontal gyrus	71	4.17	−12	42	−21
L superior occipital gyrus	116	4.1	−18	−90	33
R superior frontal gyrus	155	4.04	15	69	3
L medial superior frontal gyrus		3.76	−6	63	−6
R inferior temporal gyrus	77	3.94	54	−24	−18
R middle temporal gyrus		3.54	66	−24	−18
R middle frontal gyrus	23	3.87	39	−3	57
supplementary motor area	28	3.74	0	−6	54
L superior parietal lobule	27	3.71	−33	−69	54
R angular	137	3.69	36	−66	51
R inferior parietal lobule		3.48	39	−51	39
R supramarginal gyrus	34	3.57	63	−24	33
L precuneus	28	3.48	−3	−66	54
R fusiform	35	3.44	27	−81	−9
**Seed R thalamus Control > Patient**					
L paracentral lobule	249	5.04	−12	−15	66
R supplementary motor area	78	4.44	9	−9	54
R superior frontal gyrus	119	4.42	15	69	3
R superior parietal gyrus	213	4.29	36	−66	54
R inferior parietal lobule		4.15	48	−54	51
R middle temporal gyrus	89	4.27	69	−36	−9
L cerebellum	39	4.2	−9	−81	−21
R middle frontal gyrus	172	4.14	30	18	57
R superior frontal gyrus		4.04	15	24	54
L middle frontal gyrus	81	4.11	−36	30	48
R cerebellum	156	4.11	24	−63	−21
R fusiform		3.8	24	−72	−9
L inferior temporal gyrus	42	4.07	−57	−63	−12
L cuneus	44	3.99	−18	−78	33
R middle occipital gyrus	87	3.92	33	−69	21
R middle frontal gyrus	35	3.86	39	3	36
R precentral gyrus		2.95	39	−3	48
L precuneus	48	3.8	−9	−66	69

The MNI coordinates and t-values for the FC results. Threshold for significant clusters reported here was set at p < 0.005 (AlphaSim correction) and cluster size of 14. MNI, Montreal Neurological Institute.

### Dynamic effective connectivity variability results

**FIGURE 3:**
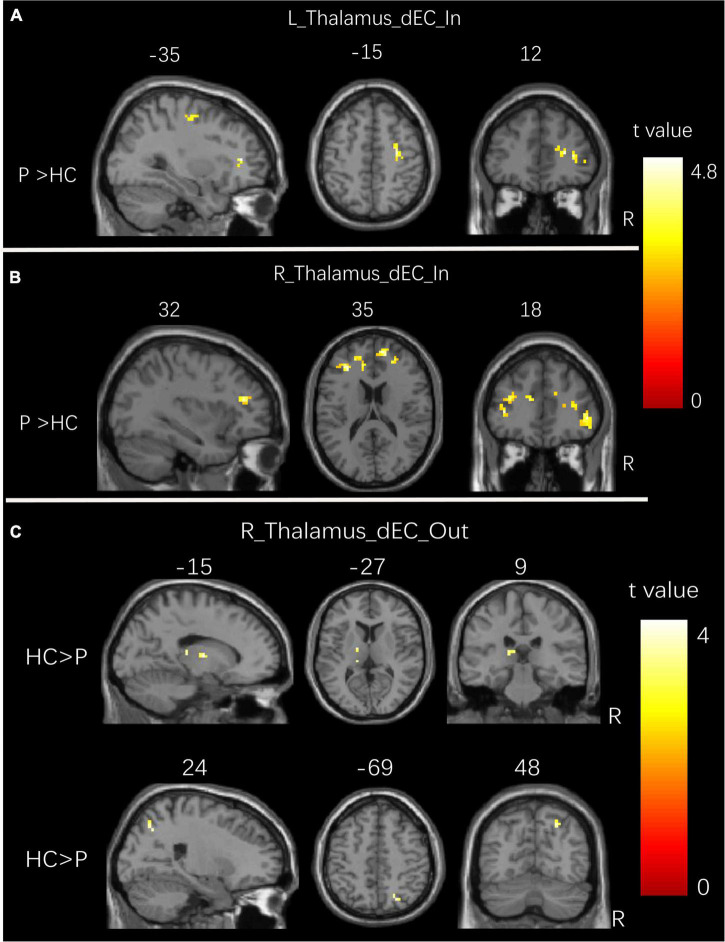
Group differences in dEC variability for bilateral thalamus seeds between two groups. **(A)** Brain regions showing significant differences in dEC variability from the whole brain to the seed of the left thalamus. **(B)** Brain regions showing significant differences in dEC variability from the whole brain to the seed of the right thalamus. **(C)** Brain regions showing significant differences in dEC variability from the seed of the right thalamus to the whole brain. The comparison analysis was thresholded at *p* < 0.005, AlphaSim corrected. R, right hemisphere; L, left hemisphere. dEC, dynamic effective connectivity; HC, healthy controls; P, patients with generalized tonic-clonic seizures.

Figure 3 and Table 4 show the significant dEC variability differences between the two groups for the bilateral thalamus seeds. Compared with the HCs, the patient group exhibited significantly excessive dEC variability from right MFG, right triangular part of IFG, and right precentral gyrus to the seed of the left thalamus (Figure 3A). Compared with the HCs, the patient group exhibited significantly excessive dEC variability from bilateral MFG and bilateral medial part of SFG to the seed of the right thalamus (Figure 3B). It is interesting that we observed a significantly low dEC variability from the seed of the right thalamus to the left thalamus and right SPL (Figure 3C).

**TABLE 4:** Significant group differences in dEC analysis.

Cluster location	Statistical values		Peak (MNI)		
	Cluster size	*t*-value	*x*	*y*	*z*
**Seed L thalamus_in Patient > Control**					
R middle frontal gyrus	21	4.43	27	45	12
R inferior frontal gyrus	25	4.35	45	39	3
R precentral gyrus	34	3.94	30	−15	51
**Seed R thalamus_in Patient > Control**					
R medial superior frontal gryus	41	4.8	12	54	18
L medial superior frontal gryus	30	3.9	−12	39	18
L middle frontal gyrus		3.85	−21	45	18
R middle frontal gyrus	42	3.68	27	45	18
L middle frontal gyrus	69	4.51	−30	36	18
L inferior frontal gyrus		4.51	−39	39	6
**Seed R thalamus_out Control > Patient**					
L thalamus	14	3.84	−15	−9	6
R superior parietal lobule	16	3.49	24	−69	48
L thalamus ^#^	8	3.23	−15	−27	9

The MNI coordinates and t-values for the FC results. Threshold for significant clusters reported here was set at p < 0.005 (AlphaSim correction) and cluster size of 14.

^#^The result was uncorrected; MNI, Montreal Neurological Institute.

### Partial correlation analysis between connectivity properties and clinical characteristics

**FIGURE 4:**
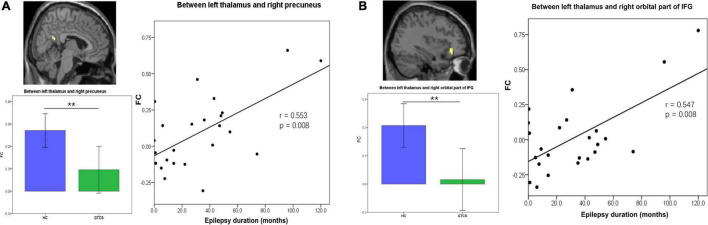
Partial correlation between static FC and the epilepsy duration in children with GTCS. **(A)** The FC values between the left thalamus and right precuneus showed a significant correlation with the epilepsy duration (*r* = 0.553, *p* = 0.008). **(B)** The FC values between the left thalamus and right orbital part of IFG showed a significant correlation with the epilepsy duration (*r* = 0.547, *p* = 0.008). FC, functional connectivity; IFG, inferior frontal gyrus.

We then further explored the potential relationship between the connectivity properties and clinical characteristics in children with GTCS. After controlling for age and sex, partial correlation analyses showed that FC between the left thalamus and right precuneus was positively correlated with the epilepsy duration (*r* = 0.553, *p* = 0.008, Figure 4A). FC between the left thalamus and the right orbital part of IFG was positively correlated with the epilepsy duration (*r* = 0.547, *p* = 0.008, Figure 4B).

**FIGURE 5:**
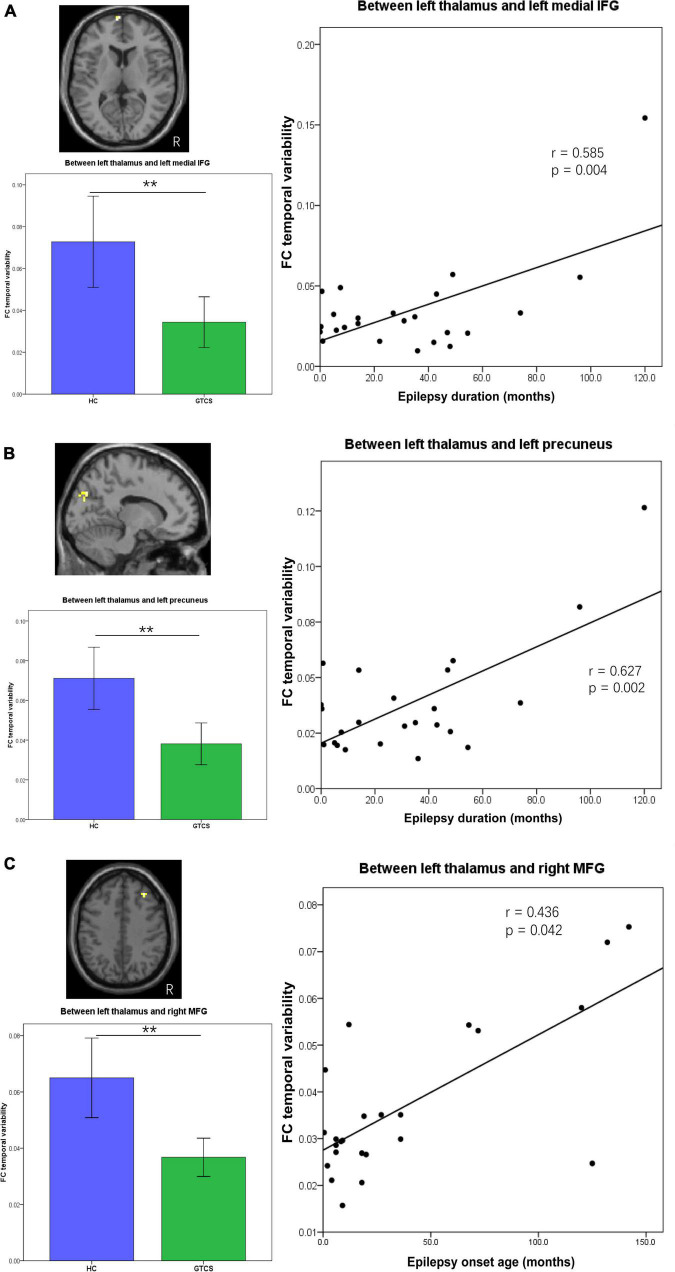
Partial correlation between connectivity temporal variability and clinical characteristics (epilepsy duration or epilepsy onset age) in children with GTCS. **(A)** The dFC temporal variability between left thalamus and left medial IFG showed significant correlation with the epilepsy duration (*r* = 0.585, *p* = 0.004). **(B)** The dFC temporal variability between left thalamus and left precuneus showed significant correlation with the epilepsy duration (*r* = 0.627, *p* = 0.002). **(C)** The dFC temporal variability between left thalamus and right MFG showed significant correlation with the epilepsy onset age (*r* = 0.436, *p* = 0.042). dFC, dynamic functional connectivity; IFG, inferior frontal gyrus; MFG, medial frontal gyrus.

The epilepsy duration of children with GTCS was positively correlated with the dFC variability between the left thalamus and left medial IFG (*r* = 0.585, *p* = 0.004, Figure 5A). The epilepsy duration of children with GTCS was also positively correlated with the dFC variability between the left thalamus and left precuneus (*r* = 0.627, *p* = 0.002, Figure 5B). Furthermore, the age of epilepsy onset in children with GTCS was positively correlated with the dFC variability between the left thalamus and right MFG (*r* = 0.436, *p* = 0.042, Figure 5C).

## Discussion

Using a seed-based connectivity approach, the current study provides a unique investigation of the static and dynamic FC of thalamo-cortical network in children with GTCS. We observed a significant decrease in static FC between bilateral thalamus and between thalamus and right inferior temporal gyrus. Dynamic FC analysis found that children with epilepsy showed decreased FC variability in the thalamo-cortical network, mostly correlated with the cortices of the frontal, motor, cerebellum, and default mode network. We also characterized the causal effect between the thalamus and the whole brain. We found increased connectivity variability from frontal gyrus to bilateral thalamus, and decreased connectivity variability from right thalamus to left thalamus and from right thalamus to right superior parietal lobe. Importantly, both static FC and connectivity temporal variability in the thalamo-cortical network showed significant correlations to the clinical features (epilepsy duration and epilepsy onset time). These alterations in static and temporal dynamic pathways connecting the bilateral thalamus exhibited the dynamic exchange of information in the thalamo-cortical network. The present findings characterize the specific role of the thalamus in children with GTCS and extend our understanding of the neural mechanism underlying the GTCS in children.

### Children with generalized tonic-clonic seizure presented decreased connection between bilateral thalamus compared with the controls

Functional connectivity is suggested to describe the relationship between the neuronal activation patterns of anatomically separated brain regions. The analysis result of this method is believed to reflect functional communication between brain regions (van den Heuvel and Hulshoff Pol, 2010). Our results indicated that children with GTCS showed a significant decrease in FC between the bilateral thalamus. The neural connection between the bilateral thalamus may also be disrupted. This view is supported by the previous neuroimaging studies in epilepsy. Patients with generalized epilepsy showed a widespread functional disruption throughout the resting state (Kim et al., 2014; McGill et al., 2014). Multiple interconnected brain systems were involved in this process, resulting in functional impairments (McGill et al., 2014; Wei et al., 2015). Previous studies in patients with GTCS found aberrant interhemispheric functional connectivity (Wang et al., 2011; Ji et al., 2014; Li et al., 2022c). In line with these findings, we observed decreased FC between the bilateral thalamus, which may indicate functional disruption between the two hemispheres. This result can reflect brain network reorganization under frequent abnormal discharges in children with GTCS.

Our study also extended results from static FC analysis to a more subtle time scale. Using the dynamic Granger causality method, we identified the flow direction and magnitude of the connection between the bilateral thalamus. This method can characterize the positive causality and negative causality between the brain regions. Given that epilepsy is a neurological disease caused by an imbalance between excitation and inhibition in the central nervous system (Goodman and Szaflarski, 2021), the causality results based on the Granger causality method may represent inhibitory and excitatory effects in physiology, which can further provide a special advantage for investigating the neural mechanism of epilepsy. Previous studies have used the Granger causality method to investigate the causal effect in epilepsy (Ji et al., 2013; Wu et al., 2015). Compared with the controls, children with GTCS showed a significant decrease in the temporal variability of the connectivity from the right thalamus to the left thalamus. The abnormal causal effect between the bilateral thalamus is unidirectional. This result is consistent with our previous studies that children with GTCS showed significant changes in spontaneous activity and gray matter volume in the right thalamus (Wang et al., 2018; Li et al., 2020b). These previous studies also found that significant correlations between the neuroimaging index and the epilepsy duration were detected in the right thalamus but not in the left thalamus. Combined with these previous studies and our present results, decreased dEC variability from the right thalamus to the left thalamus reflects the chronic damaging effect of GTCS in children. The chronic variability changes and the decreased FC between the bilateral thalamus are associated with the dysfunction of thalamo-cortical circuits in epilepsy. The specific role of the right thalamus in children with GTCS needs to be paid more attention in future studies.

### Children with generalized tonic-clonic seizure presented significant alterations in thalamo-cortical networks compared with the controls

As we know, the human brain is a complex, interconnected system with an optimal balance between functional specialization and integration. In addition, the thalamus is a cortical hub region that could integrate diverse information that is being processed throughout the cerebral cortex (Hwang et al., 2017). There is no doubt regarding the participation of the thalamus in generalized epilepsy (Lüttjohann and van Luijtelaar, 2022). Based on this prior view, we selected the bilateral thalamus as a seed to build the functional network in children with GTCS. Static and dynamic functional connectivity methods were combined in the present study. One main finding of our present study is the detection of significant alteration in the static and dynamic connections of thalamo-cortical networks in children with GTCS. As expected, decreased static FC between the thalamus and cortex (frontal and temporal cortex) was observed. This result was consistent with a previous study that showed a decreased correlation in thalamo-temporopolar connection (Bernhardt et al., 2009; Gong et al., 2021). A recent review study showed that there were overlapping findings in patients with GTCS regarding deactivation in the middle/inferior temporal gyrus (Parsons et al., 2020). Decreased thalamo-cortical connectivity was also detected between the left thalamus and right frontal pole in patients with idiopathic generalized epilepsy (Chen et al., 2021). Decreased FC between the thalamus and cognitive-related cortex in children with GTCS may render these networks less capacity to function and communicate efficiently. In clinical settings, the phenomenon of cognitive impairment in children with GTCS can be explained by these disrupted connections.

The right precuneus belongs to the DMN and is regarded as a pivotal node of the human brain network. Adults with GTCS showed a disrupted FC related to DMN (Wang et al., 2011; Kim et al., 2014). Graph theory studies showed that patients with GTCS had aberrant core hub role of regions, including precuneus and orbital frontal cortex (Li et al., 2016; Li et al., 2020b). The present study showed a significant decrease in FC between the thalamus and precuneus, which was consistent with these previous studies. The reduced FC between regions in resting-state networks may be a result of seizures in children with GTCS. Extensive FC changes in children with GTCS again verify the previous study results that seizure signal in generalized epilepsy transmit through the regions of thalamus and the bilaterally distributed brain network (Berg et al., 2010). Thus, the decreased FC between the thalamus and cortex (inferior/middle temporal gyrus, orbital IFG, and precuneus) in the present study may result from disruptions in neural connections and reflects the functional impairments of the thalamo-cortical network associated with GTCS in children.

In addition, it is worth noting that significantly different connections were found in dFC between the two groups. The decreased FC temporal variability connecting the thalamus in patients was found not only in the inferior/middle temporal gyrus, IFG, and precuneus, but also in SMA, angular gyrus, IPL, SPL, paracentral gyrus, and cerebellum. We can see that most of these regions belong to the DMN, attention, and motor network. The DMN is considered as a key network in integrating information from cognition networks (Raichle et al., 2001). Decreased static FC and dFC between the thalamus and the regions of DMN may be associated with impaired consciousness in GTCS. In addition, previous studies have detected both increased and decreased connections between the DMN and cognitive control network, which implied that deficits in self-process are correlated with cognitive function impairment in patients with generalized epilepsy (Li et al., 2017a). Despite the clear reductions in FC and significant deactivation within DMN, significantly decreased connections between DMN and sensorimotor network were observed in adults with GTCS (Liu et al., 2017; Li et al., 2022b). The findings of these previous studies implied that functional abnormality of these interconnections may influence information communication and impair functional integrations. In the present study, significant changes in the temporal variability between the thalamus and cortical regions were also detected. This result may imply that the interconnections of the brain network are disrupted in children with GTCS. As a result, the function and information communication efficiency in the thalamo-cortical network was affected, leading to cognitive impairment in patients with epilepsy.

However, the dFC results of the present study are not entirely consistent with one previous study in adults with GTCS using a similar method (Jia et al., 2020). In this previous study, significantly increased temporal variability of FC was observed both at the region level and at the between-network level. No significant changes in the dFC results were detected based on the thalamus at the region level or network level in this previous study. In the present study, we used a seed-based approach in children with GTCS to analyze the dFC and dEC of the thalamus. A significantly decreased temporal variability of FC in the thalamo-cortical network was detected in children with GTCS. Although no significantly increased temporal variability of FC was detected by the dFC method, both increased and decreased temporal variability of causal connectivity was detected by the dEC method. The temporal variability of causal connectivity from the frontal cortex to thalamus showed a significant increase in children with GTCS. Moreover, the temporal variability of causal connectivity from the right thalamus to the right SPL showed a significant decrease in children with GTCS. One explanation for the inconsistent results may be that the research subjects were different: the subjects of Jia et al. (2020) comprise adults with GTCS, while the subjects of the present study are children with GTCS. Previous studies have found that adults and children with GTCS showed a different brain organization (Li et al., 2016; Wang et al., 2018; Li et al., 2020a,b). A recent study considering the role of thalamo-cortical interaction has shown that the normal aging process can affect the interconnections between the thalamus and other brain networks (Das et al., 2021). The connection strength and direction of the thalamo-cortical networks were different between the young and old groups. The difference in the view of brain organization could also be approved by a previous study that investigated the normal development of brain white matter between healthy children and adults (Oyefiade et al., 2018). In this previous study, the participants showed significant age-related differences in diffusion index across the frontal, parietal, and temporal lobes. These age-related changes reflect continued myelination and axonal organization of short-range white matter with increasing age. Thus, the age factor may affect the connection architecture. Children with GTCS showed specific changes in the temporal properties of the thalamo-cortical network. Another explanation for this inconsistency may be the combined effect of brain development and AED in children with GTCS. Once a child has been diagnosed, AED treatment is the first choice in most cases. Then, the influence of AED on the brain is initiated. The generalized spike-wave discharge burden is moderated by AED, and the effect of the disease on normal brain development is suppressed. The combined effects of these factors resulted in this inconsistency between the children and adults with GTCS. Future studies should include both children and adults with GTCS simultaneously to verify the above explanation.

Besides, both increased and decreased temporal variabilities of causal connectivity in children with GTCS were considered to be of great interest and importance. The compensatory mechanism can explain this result. We know that the MFG and SPL belong to the central executive network (Uddin et al., 2019). A previous study in normal individuals has detected that the thalamus acts as a causal outflow hub (Das et al., 2021). The frontal cortex and superior parietal cortex were driven by the thalamus in normal individuals. In the present study, we detected increased temporal variability of connectivity from MFG to the thalamus. The increased temporal variability of connectivity implicated hyper-integration of information in the thalamus and over-interaction between the thalamus and frontal gyrus. The original balance between inhibition and excitation information through the thalamus was broken. In order to maintain the balance in the transmission of information through the thalamus, temporal variability of connectivity from the right thalamus to the right SPL was suppressed. Consistent with a previous study in idiopathic generalized epilepsy, a decreased FC between the thalamus and SPL was observed (Gong et al., 2021). Increased inhibition of this pathway may help to produce a new balance. This dynamic balance between the thalamus and regions of the central executive network can also help to maintain the critical integrative hub of thalamus. The abnormal connectivity temporal variability of thalamo-cortical network might be associated with cognitive dysfunction in children with GTCS.

### Clinical relevance of the connectivity properties in thalamus

Moreover, we observed that static FC values and dynamic FC temporal properties were significantly correlated with epilepsy duration. As shown in Figures 4, 5, epilepsy duration positively correlated with the connectivity properties of the left thalamus. Correlation analysis between the neuroimaging index and disease duration is the most popularly used method in epilepsy. Previous studies have found that disease duration was positively correlated with resting-state network abnormalities in idiopathic generalized epilepsy (Luo et al., 2012; Parsons et al., 2020; Gong et al., 2021). That is, the longer the patient had the condition, the more abnormal connections between brain regions were detected. Epilepsy duration presented negative effects on the brain connectivity in patients with generalized epilepsy (Li et al., 2016; Wang et al., 2019b; Jia et al., 2020; Xu et al., 2021). In this study, we demonstrated decreased static FC and dFC between the thalamus and brain cortex (IFG and precuneus). In the children with long epilepsy duration, their thalamo-cortical network (thalamus-IFG and thalamus-precuneus) showed a high FC or high temporal variability. That means the longer the children had the condition, the more functional connections between the thalamus and cortex regions were enhanced. This correlation result in the present study may be inconsistent with the results reported by the above previous studies. The reason for this inconsistency might be that the patient’s screening criteria were different. In the present study, all patients were diagnosed with GTCS, and the subjects with focal epilepsy generalizing secondary GTCS were excluded. Previous studies have found that these two groups of patients with GTCS demonstrated different relationships between the thalamo-cortical network connection and the epilepsy duration (Xu et al., 2021; Hsieh et al., 2022). FC between the somatosensory cortex and thalamus was negatively correlated with the epilepsy duration of focal epilepsy patients with bilateral tonic-clonic seizure, while it was positively correlated with the epilepsy duration in genetic generalized epilepsy patients (Hsieh et al., 2022). Our correlation results were consistent with this previous study. The positive correlations in the present study suggest that in the patients with the longer conditions, the alteration of connectivity between the thalamus and cortex may further serve to affect synchrony. Another reason for this inconsistency may be that the subjects of the present study were children. The combined effects of brain development and AED would help the children with GTCS to control seizures. The depressed pathways or decreased temporal variability of connections were enhanced by the effective drug treatment and normal brain development.

The relationship between the epilepsy onset age and functional properties was also calculated, as the epilepsy onset age is another factor that is known to affect the brain connectivity in epilepsy (Doucet et al., 2015). For children with GTCS, the epilepsy onset age showed a positive correlation with the dFC variability between the left thalamus and right MFG. The relationship direction is consistent with a previous study that the FC between the insular and thalamic projections was significantly correlated with the onset of illness (Gong et al., 2021). The relationship in this previous study showed that the later the onset, the lower the abnormal FC changes. A recent study also showed that the interhemispheric connectivity values within the DMN were positively correlated with the onset age of the children with GTCS (Li et al., 2022c). The brain of children with long period for normal developing would have great tolerate to the disease effect. In the present study, the dFC variability between the left thalamus and right MFG was decreased significantly. A significant correlation may imply that the children with later onset of illness would show a less decrease in the temporal variability between the left thalamus and right MFG. However, this observation needs further verification in the future.

### Limitations

Several limitations in this work should be noted, which lay the groundwork for additional important future studies. First, although information about antiepileptic medications was collected, the long-term treatment effects could not be obtained for some patients. The contact information was no longer valid. Hence, we could not combine the actual treatment effects to verify our results. Second, the sample size was relatively small, and the scanning time was short. In the future, a larger number of subjects should be recruited. In addition, more communication work with the subjects should be executed to increase the scanning time and the imaging quality.

## Conclusion

In this work, we used a combination of static and dynamic functional analyses to explore the functional properties of the thalamo-cortical network in children with GTCS. Importantly, we have shown that static FC strength and FC variability in the thalamo-cortical circuitry were decreased significantly in children with GTCS. Both increased and decreased temporal variability of causal connectivity in the thalamo-cortical circuitry was also detected in children with GTCS. In addition, the functional properties of some functional pathways showed a significant correlation with the clinical characteristics. Both increased and decreased connectivity variability in the thalamo-cortical circuitry implies a dynamic restructuring of the thalamo-cortical networks in children with GTCS. These alterations in static and temporal dynamic pathways connecting the thalamus may extend our understanding of the neural mechanism underlying the GTCS in children.

## Data availability statement

The original contributions presented in the study are included in the article/supplementary material. Further inquiries can be directed to the corresponding author.

## Ethics statement

The studies involving human participants were reviewed and approved by the Ethical Committee of the Shenzhen Children’s Hospital. Written informed consent to participate in this study was provided by the participants’ legal guardian/next of kin.

## Author contributions

YL and QC conceived and designed the experiments. QC performed the experiments. YL and XW analyzed the image data and sorted the results. QC, BQ, and JC were responsible for patient management and conceptualized the study. YL and JW wrote and reviewed the manuscript. All authors contributed to the article and approved the submitted version.

## Conflict of interest

The authors declare that the research was conducted in the absence of any commercial or financial relationships that could be construed as a potential conflict of interest.

## Publisher’s note

All claims expressed in this article are solely those of the authors and do not necessarily represent those of their affiliated organizations, or those of the publisher, the editors and the reviewers. Any product that may be evaluated in this article, or claim that may be made by its manufacturer, is not guaranteed or endorsed by the publisher.

## Footnotes

1
https://afni.nimh.nih.gov/pub/dist/doc/manual/AlphaSim.pdf

2
www.restfmri.net/forum/DynamicBC
